# Assessment of heavy oil recovery mechanisms using in-situ synthesized CeO_2_ nanoparticles

**DOI:** 10.1038/s41598-024-62393-5

**Published:** 2024-05-22

**Authors:** Nafiseh Mehrooz, Reza Gharibshahi, Arezou Jafari, Behrad Shadan, Hamid Delavari, Saeid Sadeghnejad

**Affiliations:** 1 Faculty of Chemical Engineering, Tarbiat Modares University, Tehran, Iran; 2 Department of Materials Engineering, Tarbiat Modares University, Tehran, Iran

**Keywords:** In-situ synthesis, CeO_2_ nanoparticles, Micromodel, Wettability, Oil recovery factor, Viscosity reduction, Chemical engineering, Nanoparticles

## Abstract

This project investigated the impact of low-temperature, in-situ synthesis of cerium oxide (CeO_2_) nanoparticles on various aspects of oil recovery mechanisms, including changes in oil viscosity, alterations in reservoir rock wettability, and the resulting oil recovery factor. The nanoparticles were synthesized using a microemulsion procedure and subjected to various characterization analyses. Subsequently, these synthesized nanoparticles were prepared and injected into a glass micromodel, both in-situ and ex-situ, to evaluate their effectiveness. The study also examined the movement of the injected fluid within the porous media. The results revealed that the synthesized CeO_2_ nanoparticles exhibited a remarkable capability at low temperatures to reduce crude oil viscosity by 28% and to lighten the oil. Furthermore, the addition of CeO_2_ nanoparticles to the base fluid (water) led to a shift in the wettability of the porous medium, resulting in a significant reduction in the oil drop angle from 140° to 20°. Even a minimal presence of CeO_2_ nanoparticles (0.1 wt%) in water increased the oil production factor from 29 to 42%. This enhancement became even more pronounced at a concentration of 0.5 wt%, where the oil production factor reached 56%. Finally, it was found that the in-situ injection, involving the direct synthesis of CeO_2_ nanoparticles within the reservoir using precursor salts solution and reservoir energy, led to an 11% enhancement in oil production efficiency compared to the ex-situ injection scenario, where the nanofluid is prepared outside the reservoir and then injected into it.

## Introduction

Hydrocarbon resources are one of the primary sources of energy for global industries. Consequently, researchers face challenges in sustaining production and increasing the recovery of oil from reservoirs, especially heavy and extra-heavy oil reservoirs^1,2^. In this context, the emergence of nanotechnology has played a pivotal role in the development and improvement of the oil and gas industries. Due to their unique characteristics, nanoparticles offer a promising solution for enhanced oil recovery (EOR) processes^3,4^. These particles can enhance production from oil reservoirs through various mechanisms, thanks to their expansive surface area and high catalytic activity. Nanoparticles can crack large molecules and reduce crude oil viscosity through several mechanisms included disruption of intermolecular forces, catalytic effects and thermal effects^5^. These materials can modify hydrodynamics properties, and improve the mobility ratio of the injected fluid when added to the base fluid^6,7^. Moreover, while nanoparticles can effectively penetrate the microscopic pores of reservoir rock to enhance oil recovery, careful attention must be paid to minimize potential formation damage through optimized nanoparticle size, surface charge, and injection parameters. These particles adsorb on the surface, creating a disjoining pressure^8^, consequently increasing the tendency of oil droplets to exit the pores and move toward the production well by altering the wettability of the porous medium from an oil-wet to a water-wet state^9,10^.

Surface modification or functionalization of nanoparticles is necessary to improve their colloidal stability and enhance their surface interactions^11^. This process involves introducing functional groups or surfactants to the nanoparticle surface, altering their chemical nature to make them compatible with the fluids in a system, such as oil or water. When dispersed in the fluid, these modified nanoparticles preferentially adsorb at the oil–water interface, disrupting intermolecular forces and reducing interfacial tension. This disruption leads to stabilized emulsions, as the nanoparticles form a protective layer preventing droplet coalescence^12^. Consequently, achieving industrial-scale production, economic synthesis, and a comprehensive understanding of the performance of these materials under reservoir conditions can significantly boost oil extraction from both conventional and unconventional reservoirs during field-scale operations^13^.

Nanoparticles can be produced in various ways outside the reservoir, referred to as ex-situ synthesis methods, and injected into the oil reservoir after creating a stable nanofluid^14^. Recently, researchers have turned to in-situ synthesis methods to produce nanoparticles in reservoir conditions using its energy^15,16^. In these methods, precursor salts of the nanoparticles are injected into the reservoir instead of pre-synthesized nanoparticles. The synthesis reaction takes place at the reservoir's temperature and pressure within the crude oil medium, utilizing the reservoir's energy^17^. In-situ synthesis of nanoparticles, due to the uniform distribution of particles within the porous media, significantly influences oil production mechanisms. It resolves the issue of reduced reservoir rock permeability after injection processes. Additionally, this method can enhance the economic efficiency of nanofluid injection as an EOR method since it employs precursor salts, utilizes the energy and heat available in the reservoir, and eliminates the need for special injection equipment^18^.

Conducted studies have shown that so far, a limited number of nanoparticles, including iron oxide (Fe_2_O_3_)^16,19^, nickel oxide (NiO)^18,20,21^, vanadium oxide (V_2_O_3_)^20^, alumina oxide (Al_2_O_3_)^22^, and copper oxide (CuO)^23,24^, have been synthesized in-situ for use in EOR processes. The most commonly employed synthesis method in these studies involved forming a stable microemulsion (from precursor salts) of water in oil. These nanoparticles exhibited good crystallinity and morphology, with sizes ranging from 5 to 80 nm. Typically, Parr or high-pressure reactors were used due to the high temperature required for the synthesis reaction and to provide the necessary pressure. These synthesized nanoparticles were effective in breaking down asphaltene or resin molecules and improving crude oil quality due to their favorable surface activity and proper dispersion in the oil medium. Consequently, they outperformed commercial nanoparticles in reducing oil viscosity and increasing oil production.

The size, quality, and performance of in-situ synthesized nanoparticles in EOR processes are influenced by various parameters, such as the type of precursor salt, the ionic strength of the environment, the temperature and pressure of the reaction, and the type of reservoir rock^15^. Ensuring particle properties such as size, stability, etc., at reservoir conditions through in-situ synthesis of nanoparticles involves several considerations incuding controlled synthesis conditions, real-time monitoring, tailored nanoparticle design, compatibility with reservoir fluids and characterization under reservoir conditions. By integrating these strategies, researchers can optimize the synthesis of nanoparticles in-situ to ensure desired properties for enhanced oil recovery applications in reservoir conditions.

To effectively deploy in-situ synthesized nanoparticles in field operations, future studies must provide detailed insights into their impact on oil production mechanisms within porous media. Currently, only a limited number of nanoparticles have been synthesized in real crude oil medium, and an optimal nanoparticle with the highest efficiency has yet to be identified. The investigation of how these nanoparticles affect crude oil production mechanisms, such as viscosity reduction, IFT alteration, and wettability modification, remains lacking. Although it is understood that these nanoparticles can catalyze the breakdown of heavy crude oil molecules, their influence on crude oil composition and lightening is still uncertain. The in-situ synthesis and nanocatalytic upgrading of heavy oil involves the underground decomposition of viscous heavy oil via a series of intricate chemical and physical reactions facilitated by an injected catalyst. This process allows the resulting lighter components to flow to the producer under normal pressure conditions^25^. However, further investigations are needed to understand the kinetics of these reactions and their implications for the environment and carbon production. Additionally, conducting dynamic injection tests, such as core or sandpack flooding and micromodel injection, can provide valuable insights into nanoparticle movement in porous media and their impact on crude oil recovery rates. Challenges including scalability issues, long-term stability, and formation damage persist in the field of in-situ nanoparticle synthesis. Overcoming these hurdles will necessitate interdisciplinary research efforts, innovative technologies, and collaborative initiatives aimed at advancing the application of in-situ nanoparticle synthesis for enhanced oil recovery.

While various nanoparticles have been investigated, CeO_2_ nanoparticles remain relatively understudied despite their potential to absorb and break down large organic molecules due to their catalytic and surface activity^26^. By reducing the size of CeO_2_ nanoparticles and producing them on a nanoscale, their surface-to-volume ratio increases, enhancing their catalytic activity^27,28^. CeO_2_ nanoparticles, with their ability to absorb and release oxygen through the Ce^3+^/Ce^4+^ oxidation–reduction cycle^29,30^, are particularly promising for EOR applications^31^. This project aims to fill this research gap by analyzing the impact of in-situ synthesized CeO_2_ nanoparticles on oil production. Initially, the colloidal stability of these nanoparticles was assessed. Subsequently, their impact on reducing crude oil viscosity and altering surface wettability (using carbonate rocks and glass panes) was investigated. Identification analyses were then employed to explore how the presence of these nanoparticles affects crude oil composition and lightening. Finally, micromodel injection tests were conducted to observe fluid movement within the porous medium and compare crude oil recovery between ex-situ and in-situ injection scenarios.

## Materials and method

### Materials

CeO_2_ nanoparticles were synthesized in-situ within a crude oil medium. The synthesis reaction was conducted at low temperatures by preparing a water-in-oil microemulsion. Various analyses were employed to characterize these nanoparticles, revealing their spherical shape and an average size of 16 nm. A parametric study demonstrated that the optimal synthesis of these nanoparticles, with the smallest size, was achieved by stirring a 0.55 M precursor solution at pH 10 and 65 °C for 4 h^15^. The authors detailed the synthesis procedure and characterization results in a previous paper^15^. Additionally, an Iranian heavy crude oil sample served as the reaction medium for EOR investigations, possessing properties of 19° API, a density of 0.94 g/cm^3^, and a viscosity of 1478 cP at 25 °C.

### Colloidal stability analysis

One of the critical and influential parameters when using nanoparticles in an EOR operation is their colloidal stability in the injection fluid. Nanoparticles must exhibit high colloidal stability under reservoir conditions to be used without concerns about sedimentation in pores and subsequent reductions in reservoir rock permeability, potentially leading to formation damage during the flooding process^11^. One of the primary challenges associated with in-situ synthesis methods of nanoparticles lies in accurately characterizing their long-term stability. Existing characterization techniques may lack the sensitivity or accuracy required to fully characterize nanoparticles synthesized in-situ within the reservoir, leading to uncertainties regarding their properties and behavior. The inherent black color of crude oil, coupled with the synthesis of nanoparticles within the small pores of reservoir rock, poses significant challenges to comprehensively investigating the colloidal stability of these nanoparticles using current techniques. However, the stability of the synthesized CeO_2_ nanoparticles was examined using two qualitative and quantitative methods. To achieve this, several nanofluids were prepared by dispersing varying concentrations (0.1, 0.25, and 0.5 wt%) of these nanoparticles in deionized (DI) water as the base fluid. Then, their stability was assessed by visually observing the sedimentation rate of these particles in water over a 10 days period. Also, Zeta potential analysis was conducted to measure the degree of electrostatic repulsion between particles and their colloidal stability in an aquous solution.

### Viscosity measurements

Tiny nanoscale particles possess a greater number of surface atoms due to their small size and high surface-to-volume ratio. These characteristics bestow them with favorable catalytic properties, making them suitable for use in heavy crude oil cracking processes. Their ability to reduce viscosity can enhance the movement of crude oil within porous media toward the production zone^5,26^. Consequently, this study investigates the impact of CeO_2_ nanoparticles on crude oil viscosity under various conditions. The optimal conditions obtained from Taguchi's design^15^ for the in-situ synthesis of CeO_2_ nanoparticles were employed in these tests. Table 1 outlines the designed scenarios for investigating variations in crude oil properties. The viscosity of the oil samples was measured using an Anton Paar rotational viscometer (RheolabQC model). All viscosity analyses were conducted at a shear rate of 400 s^−1^ and under ambient pressure and temperature conditions. Finally, the viscosity of each sample was calculated by averaging data collected at 100 points, with a time interval of 1 s. It is essential to clarify that to assess the reproducibility of the results, each experiment was conducted three times, and the average value of the measured viscosity was subsequently reported for each test.

**Table 1:** Designed experiments to investigate the oil viscosity variation.

Test	Conditions	Target
1	Primary oil sample without applying temperature and the presence of nanoparticles	Base test for comparison
2	Optimal conditions without the presence of nanoparticles	Finding the effect of applying temperature
3	Optimal conditions by using nanoparticles	Finding the effect of the presence of nanoparticles

### Wettability measurement

**Figure 1:**
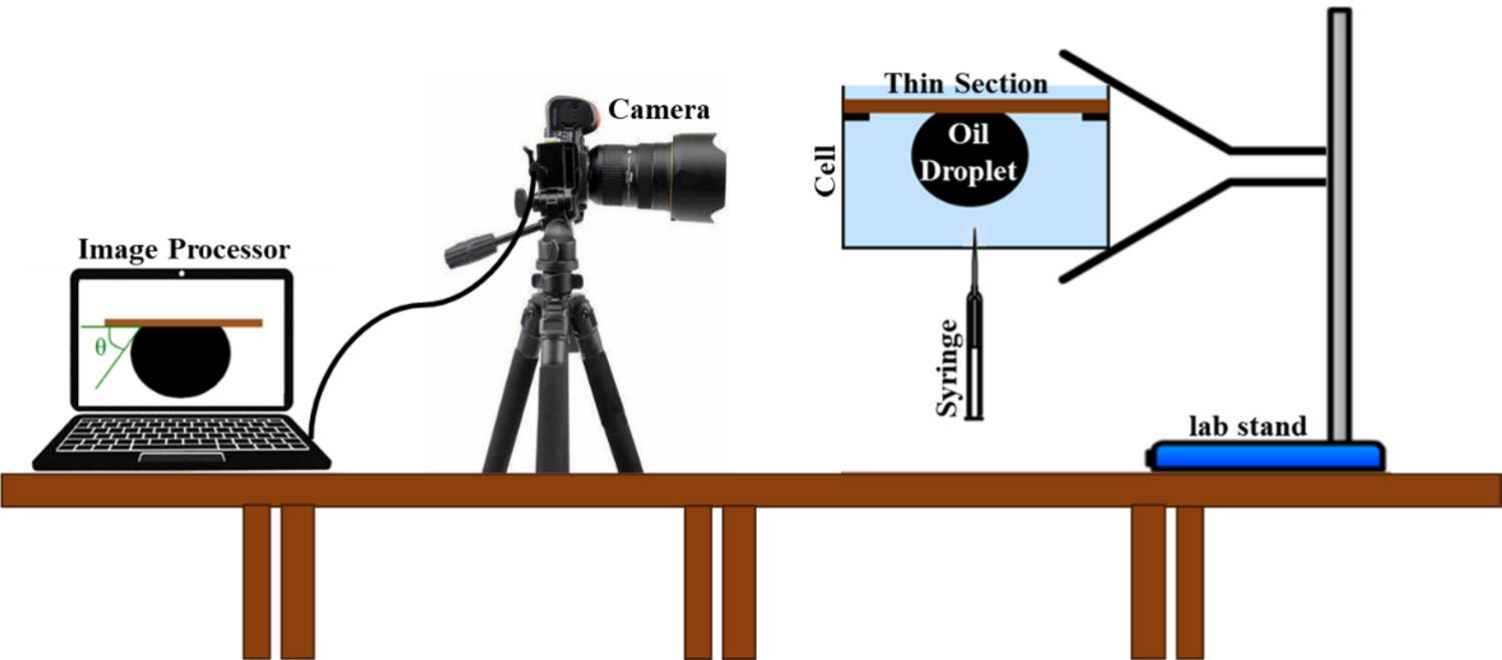
Schematic diagram of the Sessile drop method setup.

The alteration of reservoir rock wettability from oil-wet to water-wet signifies a greater inclination for oil droplets to detach from the rock surface and exit more readily from the reservoir rock pores. This factor significantly influences the effectiveness of injection processes^32^. In this research, the sessile drop method was employed to determine the oil contact angle and assess wettability alteration using the synthesized nanoparticles (Fig. 1). To accomplish this, the surfaces of certain thin sections of carbonate rocks and glass panes (measuring 3 × 2 × 1 cm) were made oil-wet^33^. Subsequently, small rock and glass pieces were immersed in various colloidal nanofluids with concentrations of 0.1, 0.25, and 0.5 wt% for 72 h at 70 °C. The analysis of wettability alteration was conducted by measuring the oil droplet contact angle at three different points on the surface of each piece, utilizing Image J software. This averaging of measurements at three points served to reduce measurement errors.

### Micromodel flooding test

**Figure 2:**
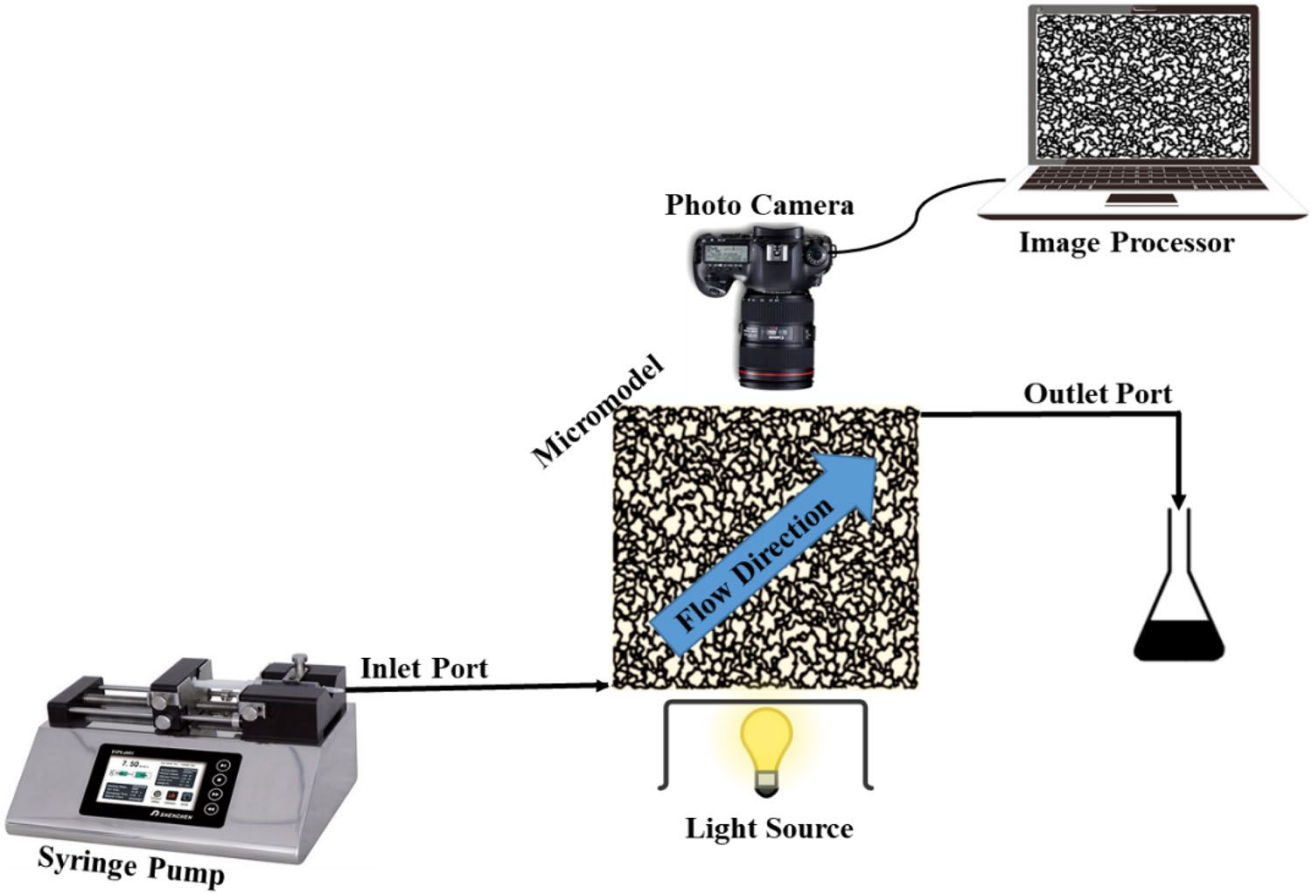
Schematic diagram of a fluid injection setup in a micromodel.

A visualization flooding method utilizing a glass micromodel flooding setup was employed to investigate and observe multiphase fluid flow within the porous medium (Fig. 2). The porous medium was represented by a transparent glass micromodel with a pattern resembling an actual oil reservoir rock. Table 2 provides the specifications of the glass micromodel used. To ensure consistency with the typical oil-wet condition found in most oil rock reservoirs, an oil-wet state was applied to each micromodel before conducting any flooding tests^11^.

**Table 2:** The characteristics of the used glass micromodel.

Pattern	Size (cm)	Depth (μm)	Porosity (%)	Permeability (mD)
Dolomite pore shape	6 × 6	60	38	890

Subsequently, the micromodels were saturated with the crude oil sample. In this project, the in-situ method of injecting nanoparticles into the micromodel was employed to explore the enhancement of oil recovery. In this approach, a solution containing precursor salts of CeO_2_ nanoparticles was initially injected into the micromodel. Following this, both sides of the micromodel were sealed, and the micromodel was placed in a thermal oven at 65 °C for 24 h to ensure complete synthesis of the nanoparticles. Finally, a syringe pump injected 0.07 ml/h of deionized water into the glass micromodel. In all flooding operations, 1 pore volume (PV) of fluids was injected. The micromodel had to be positioned horizontally to prevent gravity from affecting the results of oil production. The oil recovery factor was determined through photo analysis using Photoshop software before and after the flooding.

## Results and discussion

### Colloidal stability

**Figure 3:**
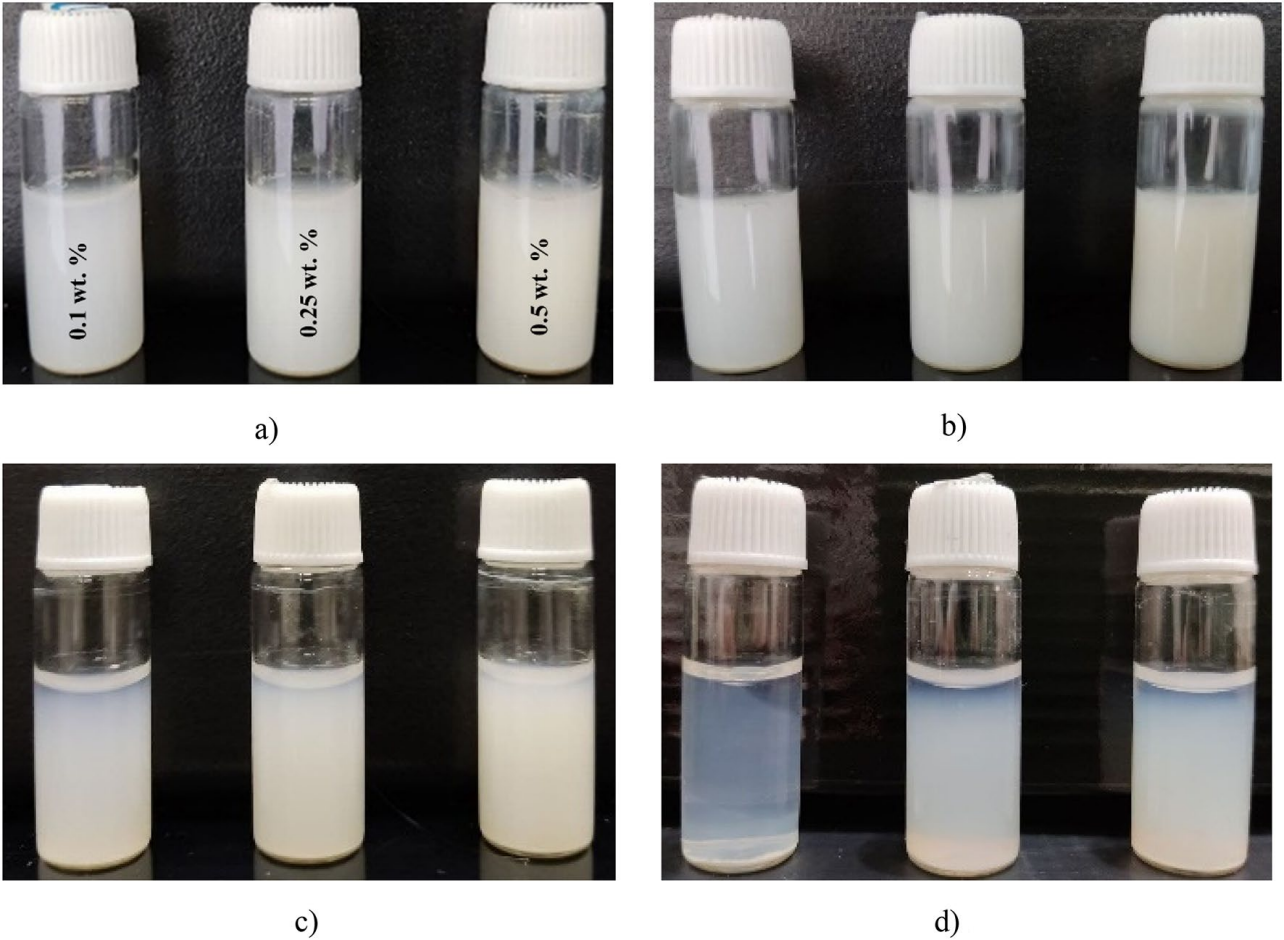
The results of the qualitative analysis of the stability of CeO_2_ nanofluids. (**a**) After 3 days, (**b**) after 1 week, (**c**) after 10 days, (**d**) after 2 weeks.

A thorough investigation into the colloidal stability of synthesized CeO_2_ nanoparticles in DI-water was conducted using both qualitative and quantitative methods. The qualitative assessment involved observing nanoparticle precipitation over a period of two weeks (Fig. 3), revealing that all prepared samples exhibited good colloidal stability for up to one week, with no sedimentation observed. However, over time (after 10 days), sedimentation within the sample containers commenced, with particles settling almost completely after approximately two weeks, indicating a loss of stability. Additionally, quantitative analysis via zeta potential measurements was performed. The zeta potential of CeO_2_ nanoparticles in distilled water can vary based on factors such as particle size, surface charge, and dispersant used. In our study, the zeta potential of synthesized CeO_2_ nanoparticles was measured at – 32 mV, indicating favorable stability in an aqueous medium. Nanoparticles with a zeta potential further from zero, whether positive or negative, typically exhibit stronger repulsion forces, hindering agglomeration and promoting colloidal stability. Therefore, a zeta potential of – 32 mV suggests significant electrostatic repulsion between CeO_2_ nanoparticles, reducing the likelihood of aggregation and enhancing their dispersion in the aqueous medium.

### In-situ upgrading of heavy crude oil

**Figure 4:**
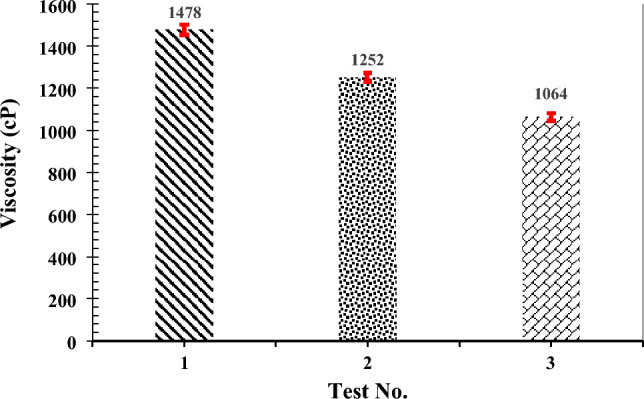
Results of the crude oil viscosity variation analysis.

Figure 4 presents the results of crude oil viscosity reduction using in-situ synthesized CeO_2_ nanoparticles for three different experiments, as outlined in Table 1.

The average initial viscosity of the primary crude oil sample, without nanoparticles and at the applied temperature (test No. 1), was 1478 cP. This test served as a baseline assessment to compare the impact of temperature and the use of nanoparticles on the viscosity alteration of the crude oil sample. The crude oil viscosity decreased to 1252 cP in test No. 2 (stirring for 4 h at 65 °C without any nanoparticles). This result demonstrates that temperature alone could reduce the crude oil viscosity by 226 cP.

In test No. 3, where CeO_2_ nanoparticles were synthesized under optimal conditions (temperature 65 °C, pH of the precursor salt solution at 10, molarity 0.55, and stirring for 4 h), the crude oil viscosity was reduced to 1064 cP. This indicates that, in addition to the temperature conditions, in-situ synthesized CeO_2_ nanoparticles could further reduce the crude oil viscosity by 188 cP. In other words, under optimal conditions, CeO_2_ nanoparticles can decrease oil viscosity by 28% (414 cP) when using the in-situ synthesis method established in this research. This outcome underscores the significant potential of the synthesized CeO_2_ nanoparticles through the in-situ method for catalytically breaking down heavy oil compounds and reducing viscosity at low temperatures (synthesis at 65 °C using the reservoir's energy)^34^. Moreover, it is important to note that without statistical analysis, it is challenging to determine the significance of the viscosity reduction and ascertain whether it is solely due to changes in that specific parameter or if other factors are involved. Therefore, a statistical analysis (t-test) was employed to determine the significance of the viscosity changes observed under each set of conditions. This analysis demonstrated that, with a confidence level of over 95%, the difference between each pair of tests can be considered significant.

**Figure 5:**
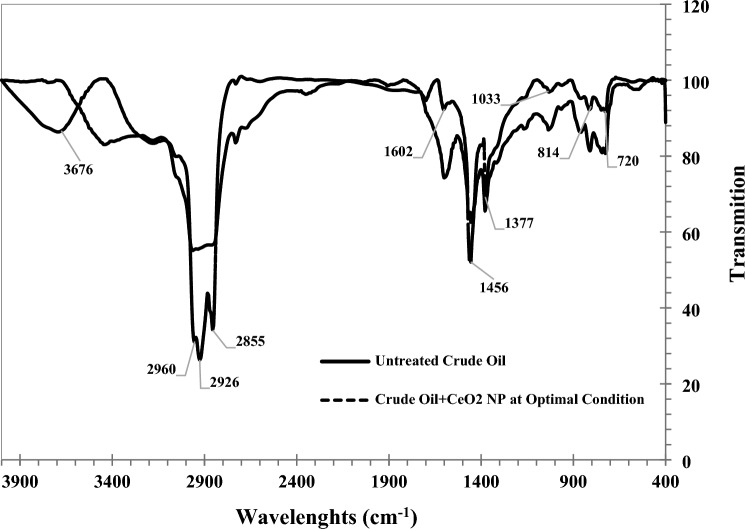
FTIR results of crude oil at 25 °C.

The proposed in-situ synthesis method from this research holds promise for EOR processes. In this state, it is possible to reduce the operation's cost, both in terms of synthesizing and injecting nanoparticles. Additionally, it offers the potential to enhance the recovery of remaining oil within a reservoir by lowering crude oil viscosity and facilitating its movement towards the production well. Fourier transform infrared spectroscopic (FTIR) analysis was conducted to investigate the ability of in-situ synthesized CeO_2_ nanoparticles to catalytically crack crude oil and enhance its quality. This analysis was performed before and after applying the optimal conditions for in-situ synthesis of CeO_2_ nanoparticles (Fig. 5).

The oil composition consists of a mixture of saturated hydrocarbons, aromatic compounds, and some complex heteroatom-containing substances. In the FTIR spectra pattern, peaks in the 2500–3700 cm^−1^ range correspond to stretching bonds of hydrogen molecules, including C–H, N–H, and O–H bonds. Peaks in the 200–2300 cm^−1^ range are attributed to triple stretching bonds, including C≡C and C≡N bonds. The wavelength range of 2000–1600 cm^−1^ pertains to double stretch bonds, including C=C, C=N, and C=O bonds. Furthermore, specific single bonds such as C–C, C–N, C–O, and C–H bonds, as well as benzene rings in the crude oil sample, are evident in the 1000–1600 cm^−1^ wavelength range. Finally, the peaks in the 400–1000 cm^−1^ range are associated with the aromatic bonds in crude oil^1,35,36^.

As observed in Fig. 5, the absorption intensity of the peaks corresponding to the symmetric (2855 ± 10 cm^−1^), scissor (1456 ± 10 cm^−1^), and rock bands (720 ± 10 cm^−1^) of –CH_2_, as well as the symmetric (2872 ± 10 cm^−1^) and umbrella bonds (1377 ± 10 cm^−1^) of –CH_3_, significantly increased when in-situ synthesized CeO_2_ nanoparticles were used. This increase suggests an augmentation of alkane chains in the crude oil sample. The crude oil became lighter due to the higher intensity of the –CH_3_ bond compared to the –CH_2_ bond. As depicted in Fig. 5, the intensity of triple or double bonds noticeably decreased, transitioning into single bonds. This transformation is one of the reasons behind the improvement in oil quality. Additionally, the absorption intensity of the aromatic bonds in the 400–1000 cm^−1^ range was reduced by CeO_2_ nanoparticles, indicating their potential to decrease aromatic compounds and asphaltenes in the crude oil sample.

CeO_2_ nanoparticles, along with other nanoparticles, such as CeO_2_, can reduce crude oil viscosity through several mechanisms. Firstly, they disrupt intermolecular forces between hydrocarbon molecules, easing their flow by weakening their interactions. CeO_2_ nanoparticles, for instance, interact with hydrocarbon molecules via van der Waals forces, facilitating viscosity reduction. Secondly, CeO_2_ nanoparticles exhibit catalytic properties that aid in breaking down larger hydrocarbon molecules into smaller fragments, a process known as cracking, thereby decreasing the oil's average molecular weight and viscosity. This catalytic activity stems from CeO_2_ nanoparticles' high surface area and active surface sites, which promote hydrocarbon molecule fragmentation. Lastly, CeO_2_ nanoparticles efficiently absorb and dissipate heat, promoting thermal cracking of hydrocarbon chains and further reducing viscosity by breaking down larger molecules into smaller ones. In conclusion, these nanoparticles have the appropriate capacity capacity to lighten crude oil and enhance its quality.

### Effect of the nanoparticles on wettability alteration

**Figure 6:**
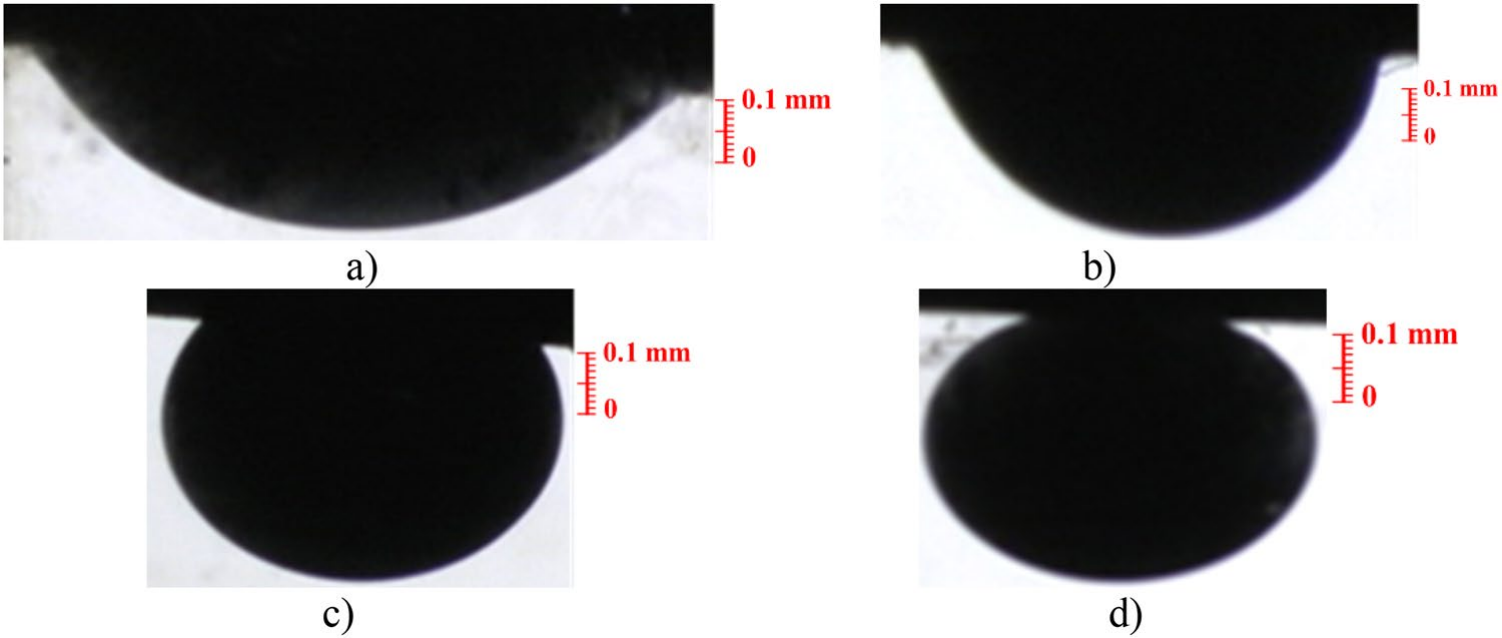
Results of wettability alteration using carbonate rock at different nanoparticle concentrations. (**a**) Water, (**b**) 0.01 wt%, (**c**) 0.1 wt%, (**d**) 1 wt%.

The study of wettability alteration in the reservoir rock was conducted by analyzing the oil droplet contact angle on the surface using the sessile drop technique. Two types of substrates, namely thin carbonate and glass sections, were employed. The surfaces of all substrates were rendered oil-wet. Subsequently, the oil droplet contact angle on these surfaces, placed in solutions with varying concentrations of CeO_2_ nanoparticles, was measured. This technique measured the apparent and static contact angle of oil droplets on the surfaces. The apparent contact angle was chosen as it provides a comprehensive assessment of the wettability alteration on the surfaces in contact with the CeO_2_ nanofluid. Also, since the droplet was in equilibrium, meaning there is no change in the shape or size of the droplet over time, the contact angle was measured statically. Figure 6 illustrates the results of the oil contact angle on carbonate sections immersed in CeO_2_ nanofluid.

**Figure 7:**
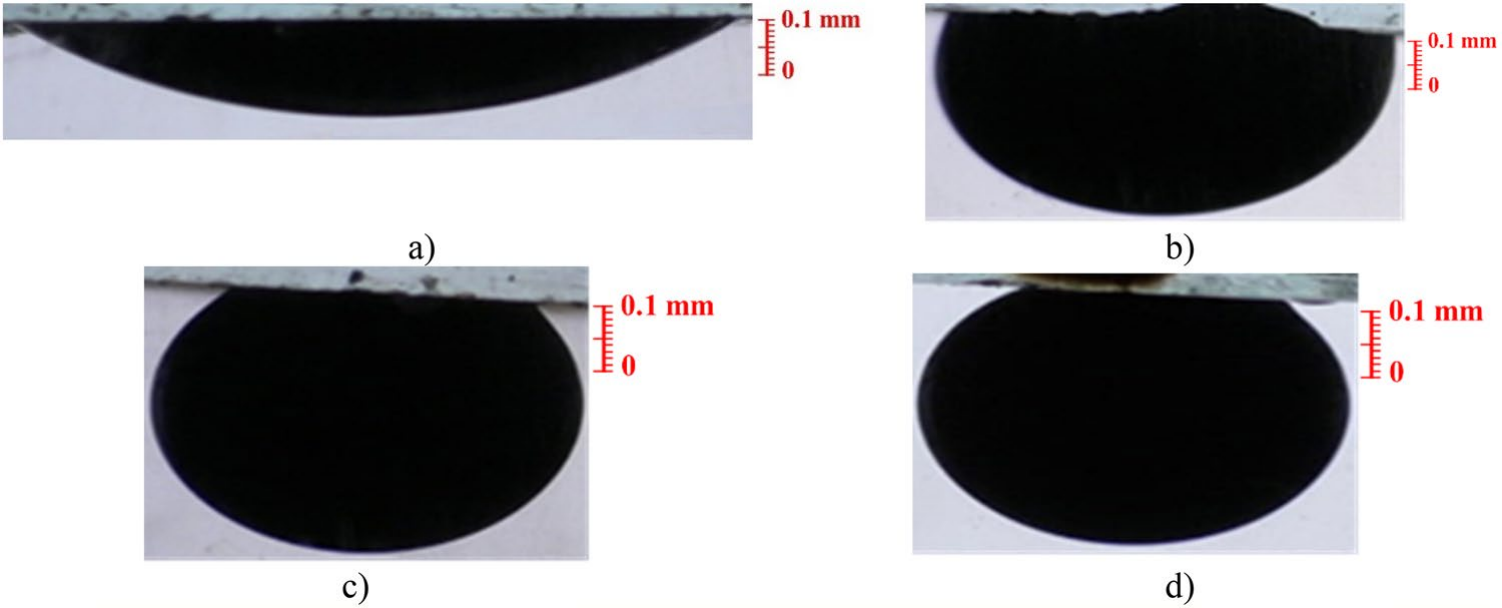
Results of wettability alteration using glass panes at different nanoparticle concentrations. (**a**) Water, (**b**) 0.1 wt%, (**c**) 0.25 wt%, (**d**) 0.5 wt%.

As shown in Fig. 6, the oil drop spread entirely on the rock immersed in DI-water, and its contact angle on the carbonate section was 141°. However, by covering the surfaces of these thin carbonate sections with different amounts of CeO_2_ nanoparticles at 0.1, 0.25, and 0.5 wt%, the oil droplet contact angle decreased to 110°, 50°, and 20°, respectively. This demonstrates that CeO_2_ nanoparticles, even in small quantities, can modify the wettability towards hydrophilicity. The surface's inclination to be covered with water improves with increasing CeO_2_ concentration. Consequently, the surface of the carbonate sections becomes completely water-wet at 0.5 wt%, owing to the high surface energy of CeO_2_ nanoparticles. Therefore, in addition to reducing crude oil viscosity, CeO_2_ nanoparticles can enhance oil production through the wettability alteration mechanism. The same experiments were repeated using oil-wet glass panes to account for the effect of substrate type on the oil drop's contact angle (Fig. 7).

The same alteration process remains evident as the substrate transitions from carbonate rock to glass panes. Initially, the oil droplet contact angle on the glass surface in a water medium was 153°. The oil-wetting of the glass panes was effectively accomplished, with the oil drop completely spreading across their surface. However, by immersing the glass surfaces in 0.1, 0.25, and 0.5 wt% CeO_2_ nanofluids, the oil droplet contact angle reduced to 80°, 43°, and 28°, respectively. Consequently, it can be inferred that irrespective of the substrate change, CeO_2_ nanoparticles exhibit significant potential to alter the surface's wettability from a hydrophobic state to a strongly hydrophilic condition.

### Effect of precursor salt injection on oil recovery factor

**Figure 8:**
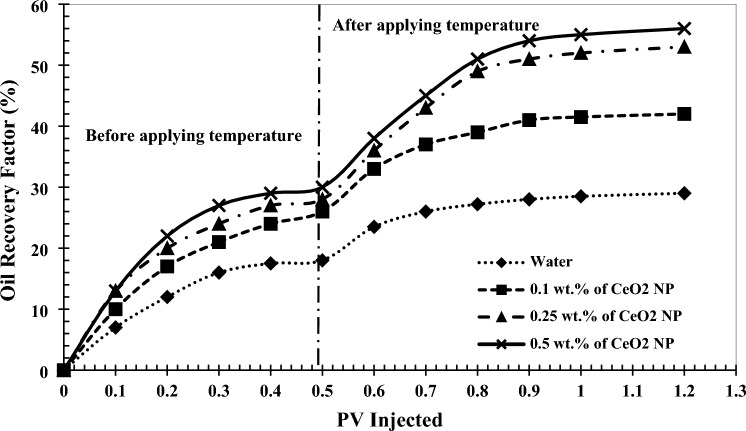
Results of oil recovery factor in micromodel flooding.

A micromodel flooding system was employed to assess the performance of CeO_2_ nanoparticles in EOR operations. Initially, DI-water was injected as a reference to investigate the influence of adding CeO_2_ nanoparticles to this fluid on oil production. Subsequently, the precursor salt solution of CeO_2_ nanoparticles at three concentrations of 0.1, 0.25, and 0.5 wt% was injected into the micromodel. Figure 8 illustrates the improvement in oil production during these four injection tests.

As depicted, the oil recovery factor after injecting 0.5 pore volumes (PV) (to ensure the breakthrough of all injection scenarios) of DI-water and the precursor salt solution of 0.1, 0.25, and 0.5 wt% CeO_2_ nanoparticles was 18%, 26%, 28%, and 30%, respectively. This demonstrates that by adding precursor salts at varying concentrations to DI-water and altering its hydraulic properties, resulting in an improved mobility ratio, the oil recovery factor increased by 8% to 12%. Furthermore, the application of temperature and the continuation of the injection process with DI-water in the water injection case increased oil production by 11%. This is attributed to the reduction in oil viscosity resulting from temperature application. Consequently, the injection fluid encounters less resistance in its path, enabling it to recover more oil from the porous medium.

**Figure 9:**
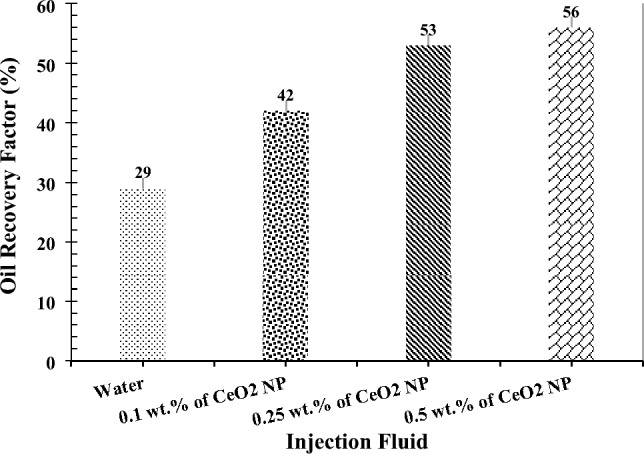
The results of the ultimate oil recovery factor (after 1 PV injection).

However, CeO_2_ nanoparticles can be synthesized in-situ by applying temperature to the porous medium containing precursor salts. In this scenario, the wettability of the porous medium transitions to a complete hydrophilic state, and crude oil viscosity decreases significantly more than with DI-water injection alone, owing to the excellent catalytic properties of CeO_2_ nanoparticles (as confirmed by the results in Sections “Colloidal stability” and “In-situ upgrading of heavy crude oil”). Therefore, the oil recovery factor increased to 42%, 53%, and 56% by continuing the injection at 0.1, 0.25, and 0.5 wt%, respectively (Fig. 9). Consequently, the final oil recovery factor demonstrated an increase of 13% to 27% when utilizing in-situ prepared CeO_2_ nanoparticles compared to water injection alone. This confirms the high potential of in-situ synthesized CeO_2_ nanoparticles for use in EOR processes. However, a thorough cost–benefit analysis is crucial for assessing the economic viability of using in-situ synthesized of nanoparticles in EOR processes. Factors like increased oil production, enhanced recovery rates, and project profitability must be considered. If the benefits outweigh the higher costs, nanofluid implementation may be justified. Advances in nanotechnology could reduce nanoparticle costs, potentially making nanofluid-based EOR techniques more economically feasible. Therefore, while nanoparticles can be expensive, their long-term economic viability should be evaluated based on reservoir characteristics, oil prices, operational efficiency gains, and advancements in nanoparticle synthesis^37^.

Additionally, it is evident that by increasing the concentration of CeO_2_ nanoparticles from 0.25 to 0.5 wt%, the oil production efficiency only improved by 3%. This is due to the increased likelihood of particle deposition and pore-clogging within the micromodel with higher nanoparticle concentrations exceeding the optimal value. Moreover, the impact of nanoparticles on mechanisms related to crude oil production from the porous medium, such as enhancing the mobility ratio, diminishes. For example, concentrations of 0.25 and 0.5 wt% have nearly the same effect on altering the wettability of the porous medium.

### The fluid flow of injected fluid into the micromodel

**Figure 10:**
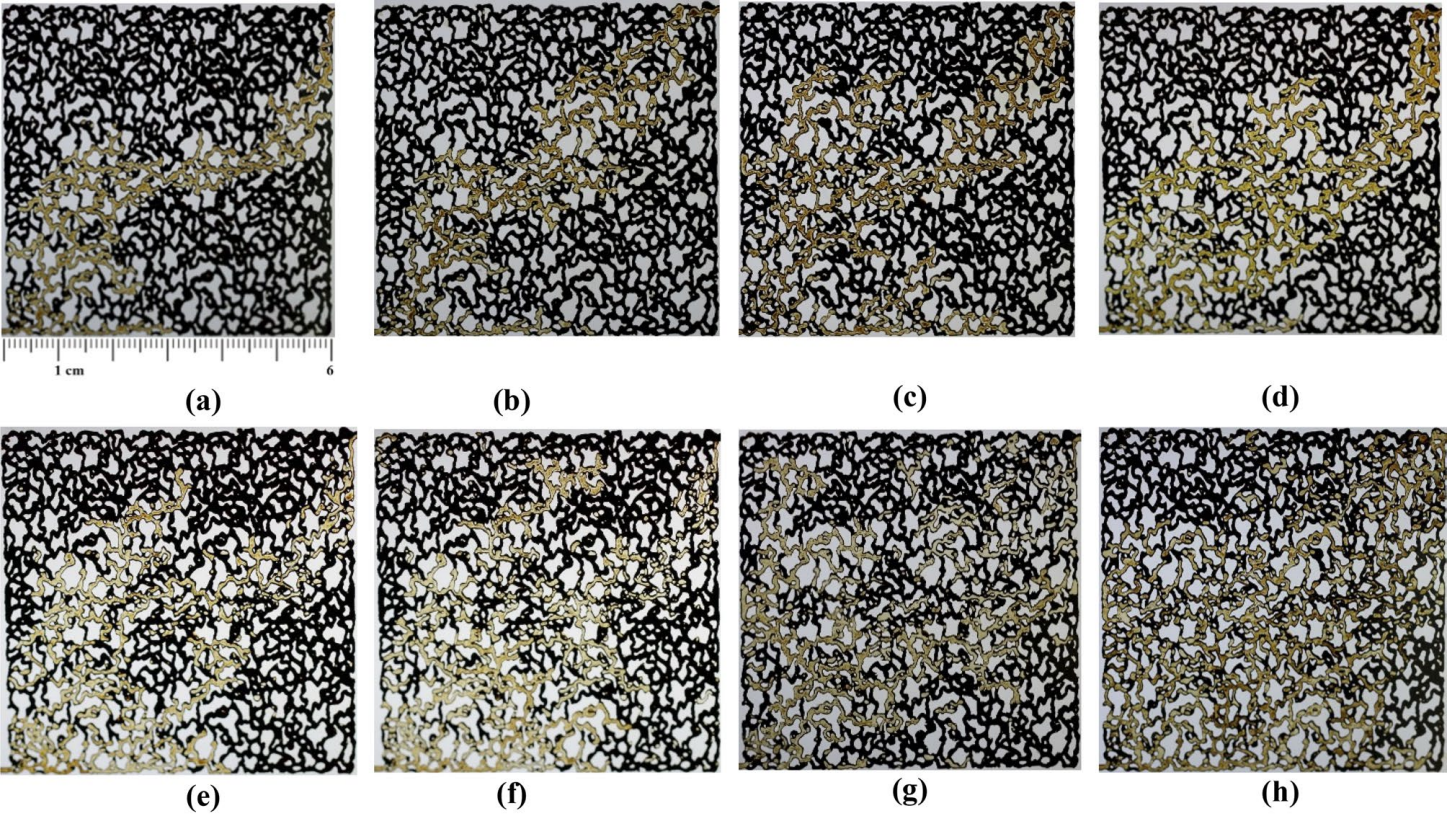
Injected fluid flow into the porous media (**a**–**d**) without and (**e**–**h**) with the application of temperature. (**a**) water, (**b**) 0.1 wt% CeO_2_, (**c**) 0.25 wt% CeO_2_, (**d**) 0.5 wt% CeO_2_, (**e**) water (**f**) 0.1 wt% CeO_2_, (**g**) 0.25 wt% CeO_2_, (**h**) 0.5 wt% CeO_2_.

One of the advantages of utilizing a glass micromodel is the ability to observe fluid movement within the porous medium. The micromodel can demonstrate fluid-surface interactions, including the deposition of nanoparticles in the pores and trapping effects within the porous medium. Figure 10 presents the flow of injected fluids during various flooding operations into the transparent micromodel, with and without the application of temperature.

**Figure 11:**
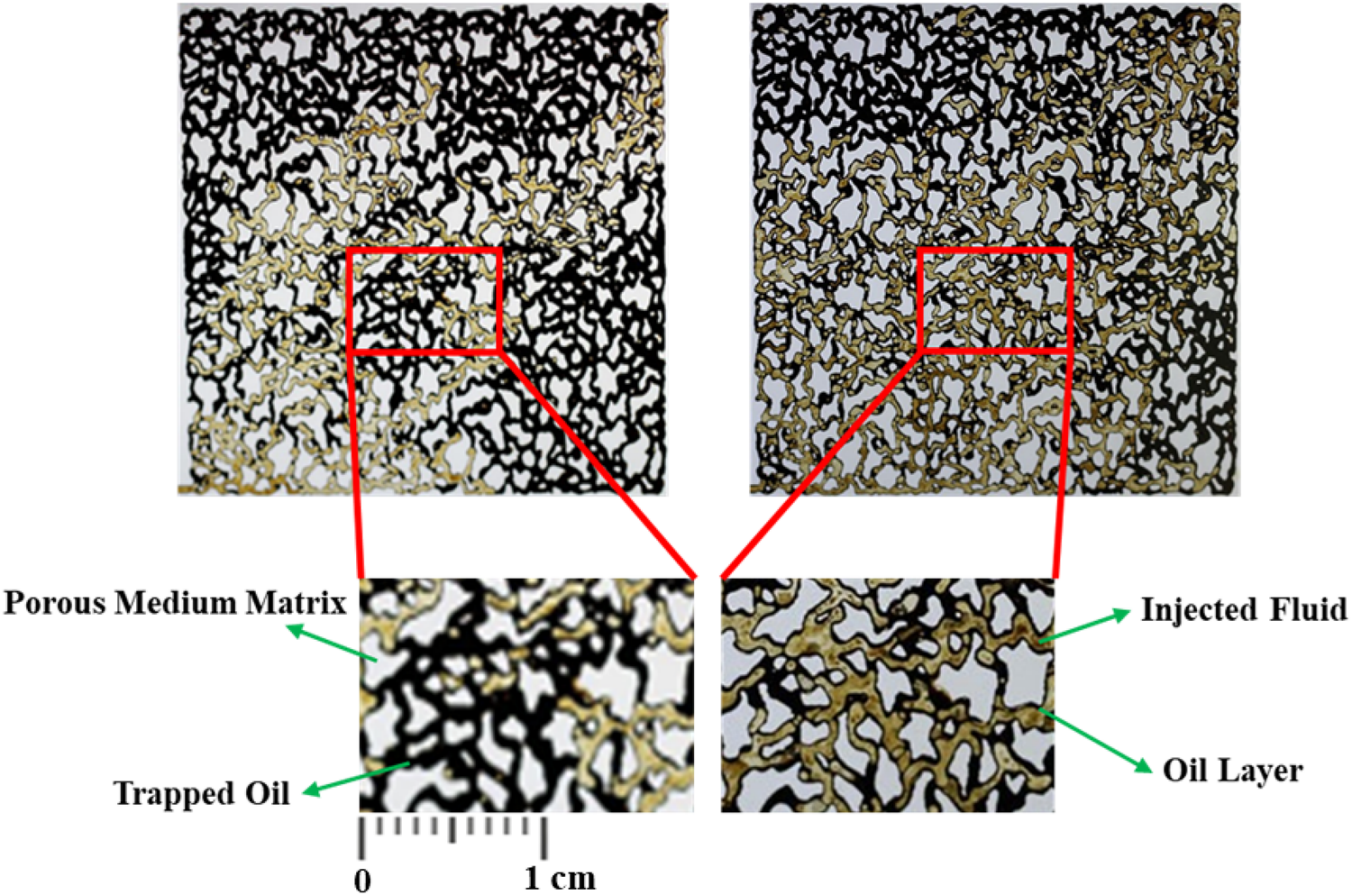
Microscopic images of (**a**) water and (**b**) 0.5 wt% of in-situ synthesized CeO_2_ nanofluid injection.

The movement of the injection fluid within the micromodel was enhanced by adding precursor salts to DI-water as the base fluid. This led to a reduction in the fingering phenomenon, allowing the injection fluid to sweep a larger portion of the porous medium. Consequently, more oil was recovered from the porous medium. After applying temperature, the fingering phenomenon decreased further. In this mode, the injected fluid recovered more oil from the micromodel compared to water injection alone, leading to a significant increase in the ultimate oil recovery factor when CeO_2_ nanoparticles were synthesized in-situ during the flooding operation. Microscopic images of a specific area of the glass micromodel were used to examine the trapping effect. Figure 11 illustrates the ability of water injection and 0.5 wt% in-situ synthesized CeO_2_ nanofluid to remove the oil layer adhering to the pores of the micromodel at the microscopic scale.

As depicted, CeO_2_ nanoparticles reduce the tendency of oil droplets to adhere to the rock surface by altering the surface wettability. This reduction in oil layer thickness on the pores of the porous medium allows the injected fluid to penetrate easily into the pores and throats, resulting in oil recovery with less oil trapped within them (consistent with Section “In-situ upgrading of heavy crude oil”).

### In-situ vs. ex-situ flooding operations to enhance oil production

**Figure 12:**
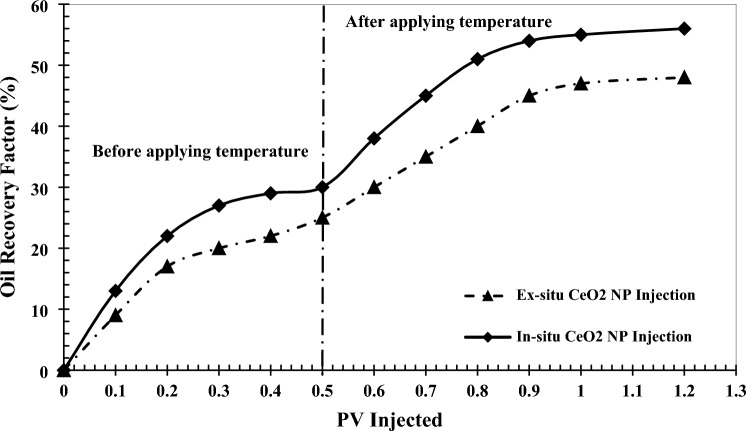
Comparison of oil recovery factor for 0.5 wt% in-situ and ex-situ CeO_2_ nanoparticles injection.

Figure 12 illustrates the effectiveness of nanoparticle synthesis using the in-situ approach compared to the conventional ex-situ synthesis method in an EOR process. The oil recovery results for the injection of 0.5 wt% of CeO_2_ nanoparticles in both in-situ and ex-situ conditions were compared.

**Figure 13:**
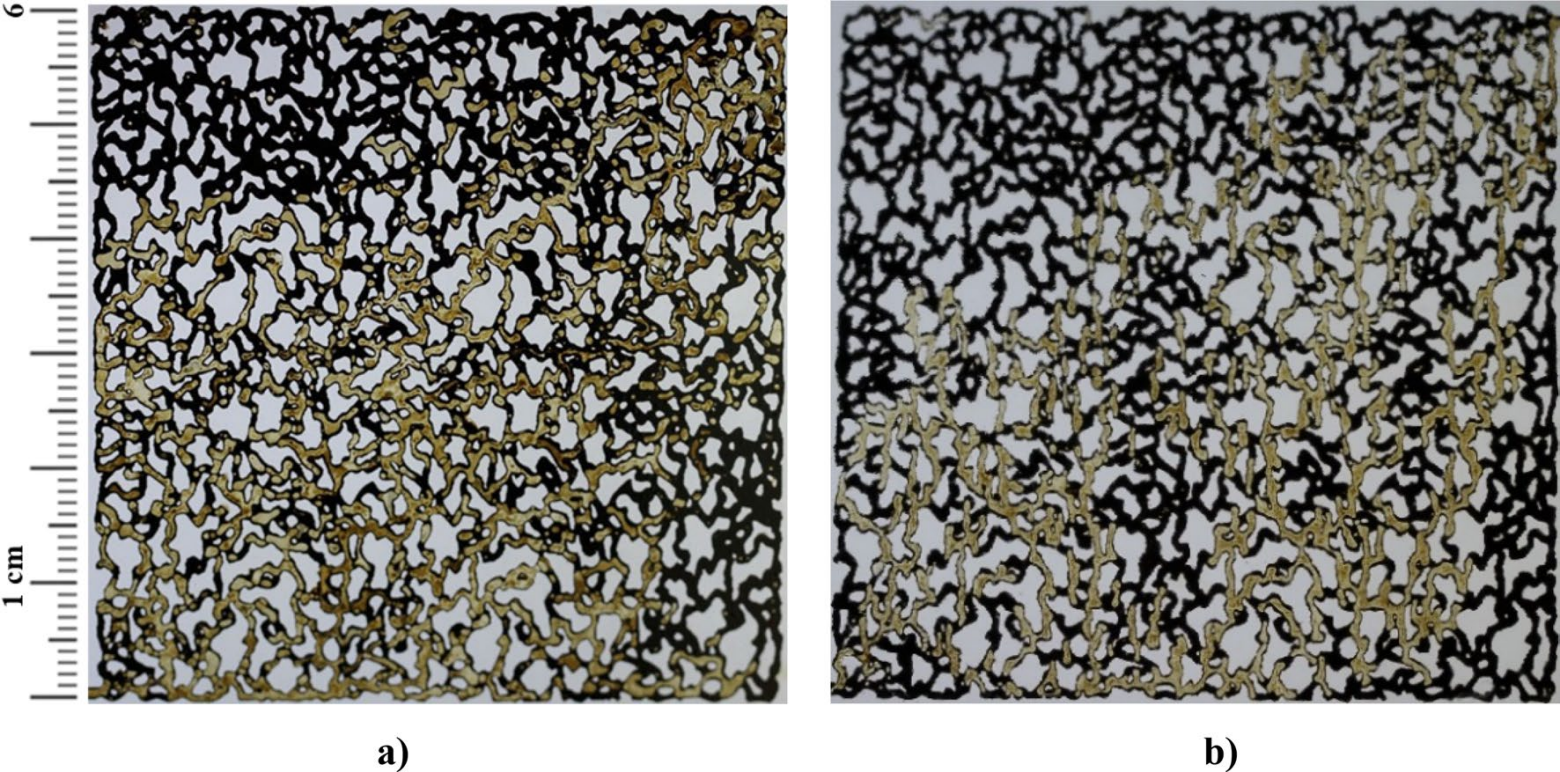
Fluid flow of injected fluid into the micromodel (**a**) in-situ and (**b**) ex-situ injection of 0.5 wt% CeO_2_ nanoparticles.

As shown, in the case of ex-situ synthesis of CeO_2_ nanoparticles, the oil recovery factor reached 45%. In contrast, the ultimate oil recovery factor at the same concentration was 56% when CeO_2_ nanoparticles were synthesized in-situ. Therefore, the in-situ injection mode of CeO_2_ nanoparticles can enhance oil production more effectively than the ex-situ injection mode, resulting in an 11% increment. This improvement can be attributed to better nanoparticle dispersion within the porous medium, reduced permeability reduction, and a more significant impact on oil production mechanisms. Additionally, Fig. 13 illustrates the fluid movement in these two injection processes, corroborating the recovery factor results.

## Conclusion

This study examined the impact of low-temperature, in-situ synthesis of CeO_2_ nanoparticles on oil production mechanisms within a transparent micromodel. The findings revealed that CeO_2_ nanoparticles synthesized via the in-situ approach could reduce crude oil viscosity by 28% (414 cP), underscoring their ability to break down large molecules and decrease oil viscosity at lower temperatures. Additionally, CeO_2_ nanoparticles altered the surface characteristics of carbonate rock and glass sections, shifting their tendency from hydrophobic to strongly hydrophilic. This shift was evident in the reduced contact angle of oil droplets to 20° at a concentration of 0.5 wt%. Flooding operations further demonstrated a significant increase in the ultimate oil recovery factor, ranging from approximately 13% to 27%, when compared to water injection alone, by utilizing in-situ synthesized CeO_2_ nanoparticles. Ultimately, it was determined that the in-situ injection mode of CeO_2_ nanoparticles exhibited greater potential for enhancing oil production, with an 11% increment, compared to the ex-situ injection mode.

## Data Availability

All data generated or analysed during this study are included in this article. Email for contact: ajafari@modares.ac.ir.
